# Minimum requirements for scientific training in medical studies

**DOI:** 10.3205/zma001740

**Published:** 2025-04-15

**Authors:** Julia Eckel, Elena Sperk, Wilko Thiele, Katrin Schüttpelz-Brauns

**Affiliations:** 1 Medical Faculty Mannheim Heidelberg University, University Medical Center Mannheim (UMM), Division for Study and Teaching Development, Medical Education Research Department, Mannheim, Germany; 2 Medical Faculty Mannheim Heidelberg University, University Medical Center Mannheim (UMM), Mannheim Cancer Center Clinical Trials Unit, Mannheim, Germany; 3 Medical Faculty Mannheim Heidelberg University, European Center for Angioscience (ECAS), Department of Microvascular Biology and Pathobiology, Mannheim, Germany

**Keywords:** medical education, scientific competence, learning objectives, standards

## Abstract

We propose a new approach to deriving minimum standards for scientific training in medical studies. This approach allows specific learning objectives to be clearly defined and presented, in an easily comprehensible manner. We contend that a fundamental prerequisite for university studies is the instruction in the systematic scientific method that can be described through the scientific cycle. This instruction provides the foundation for the acquisition of scientific knowledge and evidence-based practice in medicine.

## Abstract (de)

Wir schlagen einen neuen Ansatz zur Ableitung von Mindeststandards für die wissenschaftliche Ausbildung im Medizinstudium vor. Dieser Ansatz ermöglicht es, konkrete Lernziele nachvollziehbar zu definieren und klar darzustellen. Wir sehen die Vermittlung eines systematisch-wissenschaftlichen Vorgehens als Grundanforderung an ein universitäres Studium, das mit dem Wissenschaftszyklus beschrieben werden kann. Dieses bildet die Grundlage sowohl für wissenschaftlichen Erkenntnisgewinn als auch für evidenzbasiertes Handeln in der Medizin.

## Introduction

A sound scientific understanding is a prerequisite for evidence-based action in medicine [23], and the application of scientific findings in everyday clinical practice has a positive influence on quality in patient care [23], [24]. Physicians must therefore read scientific publications and critically evaluate the extent to which the findings presented in them, or derivable from them, have implications for their clinical practice. In reality, however, practicing physicians frequently report that they lack the skills to evaluate clinical studies or other scientific publications with regard to their quality and validity [28]. 

This proficiency gap is already apparent during their studies, where medical students do not feel adequately prepared to conduct research independently [7], [27]. Moreover, the content taught during their studies should also prepare medical students for a doctorate, which is actually intended to demonstrate their ability to conduct scientific research. Without fostering a fundamental understanding of science during training, there is a risk of a shortage of future researchers in medical science [9], [20]. This problem has already been recognised, and for years, there have been calls from various directions to improve scientific education in medical studies, not only due to the rapid growth of knowledge and technological advancements [1], [3], [4], [5], [6], [23], [21], [25], [33]. The Association of the Scientific Medical Societies in Germany (2008) [25], the German Research Foundation (2010) [6], the German Council of Science and Humanities (2014) [33], and the Association of Medical Faculties (2016) [22] have long acknowledged the necessity for the enhanced acquisition of fundamental scientific competencies throughout the course of medical studies. In addition, universities, in their role as research institutions and educational institutions for scientific skills, fear a “deprofessionalization” of medical training and the loss of the scientific basis (“de-academisation”) [25]. This is evidenced by the divergence in the content and focus of the scientific curricula of different medical faculties in Germany. While two-thirds of the innovative and reform-based degree programmes integrate a compulsory module on scientific work, this is only the case for around a quarter of the traditional degree programmes [2]. 

The German National Competency-Based Learning Objective Catalog (NKLM) [14], including the chapter on medical-scientific skills (VIII.1.), is currently being revised to reduce learning objectives and redundancies. Since the medical curricula are currently overloaded with subject-specific knowledge and learning objectives, there is critical opposition to a curriculum reform. Accordingly, discussions are ongoing at the faculties regarding how scientific thinking and research can be integrated into medical programs, raising the question of the minimum requirements for scientific rigor in medical education. At the same time, the faculties face the challenge and responsibility of sufficiently realising the scientific education within the medical curriculum as part of a university education. With this commentary, we aim to stimulate further discussion on the minimum standards for scientific education in medical studies.

## Scientific-systematic method

Scientific competence is defined as “the ability to identify problems, generate hypotheses, search for and evaluate evidence, draw conclusions, and communicate and critically evaluate scientific thinking and its results” [33] (for further information, please refer to [8] and [19]).

**Figure 1:**
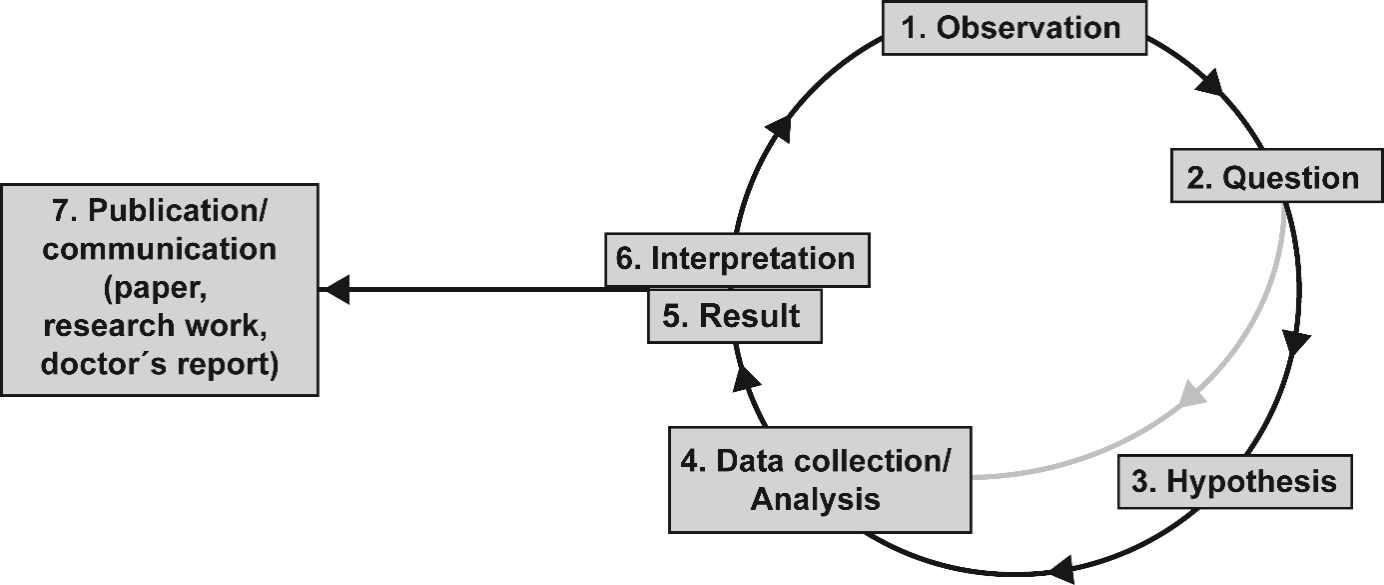
Scientific cycle: The scientific-systematic method Based on an observation that is placed in the context of existing knowledge, a question is formulated from which one or more hypotheses are derived in the hypothesis-testing procedure. In order to test the hypotheses, data is collected in a systematic manner, either diagnostically, analytically or experimentally, and then evaluated and interpreted. This process enables the research question to be answered and the results to be integrated into existing knowledge. The results and conclusions may be communicated in a variety of formats, including presentations, scientific publications (papers), research works, or even guidelines, doctors' reports, or patient consultations. The knowledge gained in this way frequently represents an observation in itself or gives rise to new observations that result in the inception of a new scientific cycle. In the event that the available data is insufficient, it may be necessary to undertake an exploratory, hypothesis-generating procedure prior to the formulation of hypotheses (grey arrow). This involves the systematic collection of data based on the research question without the initial formulation of hypotheses, which are then subjected to analysis and interpretation. The data thus collected represents an observation in itself, and serves to supplement the original observation, thereby enabling the research question to be specified with greater precision. This, in turn, allows the formulation of hypotheses and the commencement of a hypothesis-testing procedure in the next phase of the cycle.

Scientific competence is therefore the ability to apply the scientific method that can be described with the help of the scientific cycle (see figure 1 (Fig. 1)).

The scientific method has its origins in the work of Hippocrates of Kos (460-370 BC) and encompasses abductive, retroductive, deductive and inductive reasoning and argumentation [19]. Although the specific starting points and the emphasis on individual steps of the scientific cycle may vary across different disciplines [8], the method is generic, meaning it is the same in all areas of medical research and across disciplines (see attachment 1 ).

In their everyday clinical practice, medical doctors must also adopt a scientific and systematic method, which involves developing questions, generating hypotheses, collecting data and deriving, documenting and communicating evidence-based working strategies. The critical analysis of a paper in everyday clinical practice, or in the context of research, also requires checking whether scientific standards have been adhered to in the individual steps of the scientific cycle and whether the statements in the paper are valid. 

## Derivation of the minimum requirement for scientific training

The minimum scientific training requirement for medical students can be clearly derived from the scientific cycle as the overarching structure. It is defined as the ability to independently conduct scientific and systematic inquiry, utilising the individual steps outlined in figure 1 (Fig. 1). This ability serves to prepare for and implement a doctorate, enabling the independent conduct of studies aimed at generating knowledge in various research areas (with their respective discipline-specific research methods) and the development of guidelines and subsequent targeted application of evidence-based medicine according to the current state of scientific knowledge. Table 1 (Tab. 1) shows the thus derived science-oriented learning objectives for a future core curriculum of medical faculties, depending on the competencies required for the scientific-systematic method.

It is essential that the learning objectives are operationalised in the curriculum at both the individual level (e.g. literature research) and within a contextual framework (as part of the scientific-systematic method). In order to assess scientific competence at the final examination stage, students should be required to complete a scientific paper independently, under the guidance of a supervisor. The necessity for independent performance in scientific work has been discussed at length and justified in terms of learning theory. It is also pertinent to cite the ongoing debate surrounding research-based teaching (see references [12], [15], [16]), Holzkamp’s (1995) subject-science method to learning (see [17]), and the fundamental concepts of research-based learning as outlined by Huber (2009) (see [18]). Furthermore, it is of great significance to establish a connection between scientific discoveries, and their impact on clinical practice. This can for example be achieved by examining the applicability of current scientific evidence to patient cases. 

## Conclusion

It is imperative that scientific skills be taught at medical faculties in Germany, as they form the foundation for scientific inquiry and medical care, with consideration of the current state of scientific knowledge. In this context, the facilitation of the aforementioned systematic scientific method, from which the science-oriented learning objectives formulated for the core curriculum can be derived, represents a fundamental prerequisite for a university-level education. 

## Authors’ ORCIDs

- Elena Sperk: [0000-0002-8771-8124]
- Wilko Thiele: [0000-0002-8978-4192]
- Katrin Schüttpelz-Brauns: [0000-0001-9004-0724]

## Competing interests

The authors declare that they have no competing interests. 

## Supplementary Material

Illustrative examples of the application of scientific-systematic procedures in the context of preclinical, clinical, and health services research, as well as in the context of a clinical case

## Figures and Tables

**Table 1:**
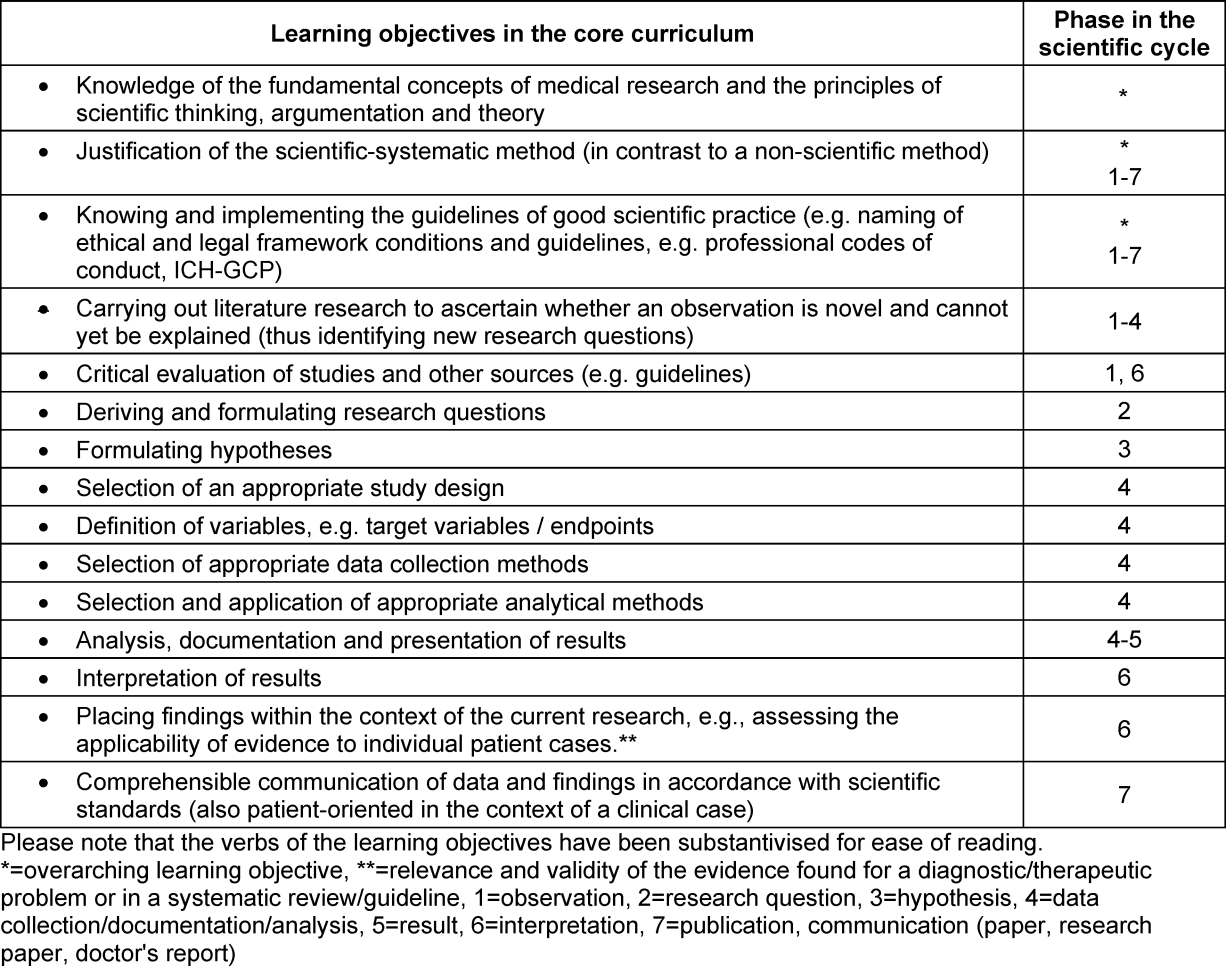
Derived science-oriented learning objectives for a prospective core curriculum for medical faculties
