# Supplementary material for: Minimum requirements for scientific training in medical studies
Source: GMS J Med Educ. 2025 Apr 15;42(2):Doc16. doi: 10.3205/zma001740 (PMC12131508; doi:10.3205/zma001740)
Supplement: Illustrative examples of the application of scientific-systematic procedures in the context of preclinical, clinical, and health services research, as well as in the context of a clinical case [file JME-42-16-s-001.pdf]

Attachment 1: Illustrative examples of the application of scientific-systematic procedures in the context of preclinical, clinical, and health services research, as well as in the context of a clinical case

These examples serve to illustrate the generic nature of the scientific-systematic method and show examples from preclinical research, clinical research and healthcare research. The evidence-based approach is also presented in the context of an exemplary clinical case.

Context	Observation	Question	Hypothesis	Data collection/ analysis	Result	Interpretation	Publication
**Preclinical research**	Ketogenic diets can improve the well-being and quality of life of breast cancer patients. However, the precise impact of this dietary approach on tumour growth and metastasis remains inconclusive, with evidence suggesting both beneficial and detrimental effects.	Does a ketogenic diet exert an influence on the growth and metastasis of breast tumours?	A ketogenic diet may influence the growth and metastasis of breast tumours in a mouse model.	Mice that develop breast tumours as a result of genetic alterations are randomly assigned to either a ketogenic diet or a standard diet (control group). The volume of the tumours and the number of lung metastases are quantified.	The analysis revealed no statistically significant differences in tumour growth and the number of lung metastases between the group that received the ketogenic diet and the control group that received the standard diet.	The ketogenic diet has no influence on tumour growth and metastasis in the mouse model used. This suggests that breast cancer patients can likely benefit from the positive influence of a ketogenic diet on well-being and quality of life without concern for potential negative effects in terms of oncological safety.	[13]

Context	Observation	Question	Hypothesis	Data collection/ analysis	Result	Interpretation	Publication
**Clinical study**	Observation 1:<br>S3-LL: Following breast-conserving surgery, the entire breast should be irradiated. It should be noted that radiation can have side effects. A review of the literature indicates that breast carcinomas recur locally in the tumour bed in over 90% of cases. Intraoperative radiotherapy (IORT) could be a simple and rapid option for a precise and exclusive tumour bed irradiation. However, there is currently no randomised controlled trial (RCT) on IORT compared to whole-breast irradiation.	Research question 1:<br>Is local irradiation of the tumour bed alone using intraoperative radiotherapy (IORT) equivalent to standard therapy (whole-breast irradiation) for patients with a very low risk of recurrence?	Hypothesis 1:<br>Partial breast irradiation using intraoperative radiation therapy (IORT) is comparable to whole-breast irradiation in patients with a low risk profile in terms of local control after five years.	Data collection/analysis 1:<br>Randomized phase III study: TARGIT A experimental arm (IORT) vs. standard therapy (whole-breast irradiation); primary endpoint: local control after 5 years, total 10-years follow-up period for all other endpoints	Result 1:<br>Using IORT for irradiation is not an inferior approach regarding local control after 5 years compared to whole-breast irradiation; it is, in fact, significantly more effective in terms of non-breast cancer-associated survival.	Interpretation 1:<br>Transfer of the new findings to the S3-guideline, AGO recommendations; There is a survival advantage and this is even significant for non-breast cancer-associated survival. CAVE: the study was not powered to show survival effects. In addition, the patients appear to develop fewer metastases.	[30], [31], [32]
	Observation 2 (derived from the findings of the 1st partial study):<br>The high single dose administered with IORT directly after tumour resection appears to have a positive effect on tumour control outside the tumour bed, as evidenced by a reduction in the incidence of metastases. Furthermore, there is a positive effect on survival.	Question 2:<br>Does IORT have an impact beyond the tumour bed?	Hypothesis 2:<br>Patients who undergo IORT demonstrate a reduction in the incidence of metastases and exhibit superior survival outcomes compared to those who do not receive IORT, even if they experience a local recurrence.	Data collection/ Analysis 2:<br>Data from TARGIT A with subgroup analyses (biological factors) on oncological outcome	Result 2:<br>IORT patients with a tumour grade 1 or 2 differentiation, show the best survival com-pared to all other subgroups. Addi-tionally, after developing a local recurrence, IORT patients have less metastases and a significantly better breast cancer-specific and over-all survival com-pared to those who undergo standard radiotherapy.	Interpretation 2:<br>IORT has been demonstrated to exert beneficial effects in regions beyond the irradiated volume (tumour bed).	[29]

Context	Observation	Question	Hypothesis	Data collection/ analysis	Result	Interpretation	Publication
**Clinical case**	The patient presents with an extensive breast carcinoma. The examination revealed a special form of breast carcinoma, namely a “cancer en cuirasse”. The consensus of the interdisciplinary tumour board was that there was no sufficient in-house expertise on this rare form of breast cancer and that a literature search was necessary, given that the S3-LL did not provide any specific information.	Question: What is the optimal treatment plan for this patient?		Systematic review of the literature using a variety of sources. Review and evaluation of the literature according to relevant recommendations on evidence-based medicine (EBM)	Overview of treatment options for comparable patient populations, including the advantages and disadvantages	Selection of the appropriate treatment based on a close consultation with the patient, considering individual wishes and needs (shared-decision-making). After the treatment, the success of the therapy and the procedure are evaluated.	
**Healthcare research**	In the event of an elevated risk of local recurrence in breast cancer patients, it is reasonable to consider an intensified radiation dose in the tumour bed, in accordance with the recommendations set forth by S3-LL and AGO. This can be achieved through a range of techniques, including IORT. While there are several retrospective studies on this technique, including larger and smaller studies, there is currently a lack of prospective data. Given that the technique is already a standard method in the S3-LL, an RCT may not be the most appropriate approach. Instead, a prospective registry for quality assurance could be a valuable tool.	Question: Is IORT as a boost method a well-tolerated and effective treatment option for patients with a higher risk of recurrence?	Hypothesis: IORT as a boost represents a locally effective and well-tolerated form of therapy.	Prospective registry with 10 participating centres in Germany, including 1133 patients with up to 10 years of follow-up treated in a real-life setting. This means that the centres treat the patients according to their routine. Data are collected via standardised clinical report forms (CRFs) at defined time points.	Local control was excellent, as was the overall survival rate. There were only a few instances of metastasis and the toxicity profile was within the expected range.	The quality assurance of IORT as a boost has been achieved with prospective data from a large patient registry. The retrospective data from the S3-LL and the AGO recommendations have thus been validated with data from routine care.	[10], [11], [26]

Abbreviations: S3-LL=Evidence-based Guideline for the Early Detection, Diagnosis, Treatment and Follow-up of Breast Cancer, IORT=Intraoperative Radiotherapy, RCT=Randomized Controlled Trial, AGO=Arbeitsgemeinschaft Gynäkologische Onkologie (Working Group Gynaecological Oncology), EBM=Evidence Based Medicine, D=Germany
